# Experiences, Perspectives, and Professional Development Needs of Mental Health Nurses Caring for Women During the Perinatal Period in Acute Mental Health Settings

**DOI:** 10.1177/08445621261483299

**Published:** 2026-08-30

**Authors:** Sara Lamoureux, Elizabeth M. Forster, Amy E. Mitchell

**Affiliations:** 1 School of Nursing & Midwifery, 110927 Griffith University – Gold Coast Campus, Southport, Australia; 2 School of Nursing & Midwifery, 110927 Griffith University – Nathan Campus, Nathan, Australia; 3 School of Nursing, Midwifery and Social Work, 1974 University of Queensland, Brisbane, Australia

**Keywords:** Moral distress, mental illness/psychiatry, nurse/nursing, acute inpatient, pregnancy

## Abstract

**Background:**

High-quality management of mental illness for women admitted to acute mental health settings during the perinatal period is crucial due to the complexities associated with concurrent mental illness and pregnancy/childbearing.

**Purpose:**

This research aimed to identify perspectives and education needs and experiences of moral distress among psychiatric nurses caring for perinatal women in acute mental health settings.

**Methods:**

A convenience sample of 12 nurses from acute mental health units in public tertiary hospitals in south-east Queensland, Australia, completed a demographic survey and semi-structured interviews which were recorded, transcribed, and analysed using thematic analysis.

**Results:**

Four overarching themes included: (i) Nursing Care, (ii) Work Environment, (iii) Workflow Challenges, and (iv) Moral Distress. Despite potential risks for women admitted to acute mental health units during the perinatal period, nurses provided compassionate care, demonstrated advocacy, reflected upon challenging conversations, and were cognizant of patient safety. Nurses reported limited educational preparation around perinatal mental health, and perceived deficits in continuing professional development opportunities and resources. Negative feelings associated with moral distress, including worrying and struggling, were highlighted by nurses and impacted care provision.

**Conclusion:**

Nurses require support to successfully navigate providing care to this complex clinical group within equally complex health care environments, team dynamics and moral/ethical considerations.

## Background

The perinatal period, the time ranging from conception until one year after birth, is a high-risk time for developing mental illness which may initiate or change pre-existing mental health treatment (Friedman et al., 2018). Pregnancy can exacerbate psychiatric symptoms and hormonal level changes can impact drug metabolism (Friedman et al., 2018). Social factors can also impact maternal mental health including minimal social supports, poor self-esteem, intimate partner violence and unplanned pregnancy (Porter et al., 2019). If left untreated, mental illness during pregnancy can lead to adverse health outcomes for both baby and mother, including preterm birth, infants small for their gestational age, and maternal suicide (Ben-Sheetrit et al., 2018).

High-quality and multidisciplinary clinical management of pregnant women with mental illness is crucial for good outcomes for mothers and babies (Frayne et al., 2019). Pregnant and/or postpartum women may receive care outside of dedicated maternity units and admissions to other acute care units present multifaceted challenges to the nurses providing care (Bayes & Ewens, 2016). When not receiving care in the maternity units, Mother and Baby units (MBU) are considered gold standard for mothers and their babies when psychiatric inpatient treatment is required during the postnatal period (Adhikary et al., 2024) and to facilitate mother–infant relationships (Howard et al., 2022). However, globally, there is a geographical inequity in accessing mother and baby units (Howard et al., 2022). Nurses employed outside of these units may lack experience, knowledge and confidence in providing care for pregnant and/or postpartum women, and may fear harming the patient due to a lack of education, professional support, guidance and mentorship related to providing care to this community (Bayes & Ewens, 2016). In Australia, pre-registration nurse education delivers a comprehensive education model that aims to prepare registered nursing graduates capable of working competently and safely within the clinical setting (Hazelton et al., 2024). Furthermore, within this generalist education model, sexual and reproductive health is often spread across several courses with limited depth (Shi et al., 2025).

Through the course of their work, nurses encounter situations that require ethical decisions that conflict with their own values and may feel limited by constraints beyond their control, including organizational policies, workplace culture, and/or lack of resources (Johnstone & Hutchinson, 2015). These can prevent the nurse from acting morally and force them to work within the status quo, resulting in inaction or engaging in behaviours they believe are wrong (Johnstone & Hutchinson, 2015). Ethical situations may necessitate nurses choosing between patients’ autonomy, the nurse's own interests and moral integrity, and professional survival (Johnstone & Hutchinson, 2015).

### Moral Distress

The term “Moral distress” is subject to a lack of conceptual clarity with widespread causes and phenomena (Morley et al., 2019). Moral distress was initially (Helmers et al., 2020) defined in Jameton's 1984 book *Nursing Practice: The Ethical Issues* “as a challenge that arises when one has an ethical or moral judgment about care that differs from that of others in charge” (Jameton, 2013, p. 298). For the purpose of this study, Jameton's original definition of moral distress will be used.

Moral distress is experienced the moment the individual is constrained from “doing the right thing” by either internal or external circumstances (Campbell et al., 2016). Moral distress experienced by nurses has been identified as a significant issue, influencing nurses in all aspects of the healthcare system, and in turn, threatening nursing integrity and quality patient care (Johnstone & Hutchinson, 2015). Moral distress can illicit emotional responses including anger, frustration, anxiety, guilt, stress, and sense of failure (Ghafouri et al., 2021; Johnstone & Hutchinson, 2015). Physical responses to moral distress may also present, including crying, decreased appetite, tachycardia, headaches, tremors, gastrointestinal disorders, insomnia, and nightmares (Caram et al., 2022). Moral distress is associated with high rates of staff turnover, decreased job satisfaction, feelings of burnout and insecurity (Ghafouri et al., 2021), and adversely affects employee retention (Fourie, 2015). Nurses experiencing moral distress are at increased risk of leaving their positions and the profession altogether (Johnstone & Hutchinson, 2015).

Within healthcare, researchers have examined moral distress across a variety of clinical disciplines and environments (Helmers et al., 2020), including haemodialysis (Kim et al., 2023), oncology (Eche et al., 2023), and neonatal intensive care (Rezaei et al., 2023). Nurses employed in mental health settings may experience moral distress differently than nurses in other clinical settings due to restrictions placed on patient freedoms such as restraints, involuntary hospitalizations, and Consumers living with severe mental illness (Ghafouri et al., 2021). However, little is known about the moral distress nurses in mental health settings encounter (Ghafouri et al., 2021).

The aim of this research was to explore the experiences, perspectives, and professional development needs of mental health nurses when caring for pregnant and postnatal women in acute mental health settings, including experiences of moral distress in providing care during this period. Questions included focussed on the care needs of this community, resources available to mental health nurses to provide necessary care, nursing education related to pregnancy, and the impact on the nurse in providing care during the pregnancy period within acute mental health units.

## Methods

### Design

Constructivist grounded theory was used during the interview process as it allowed for an inductive, iterative, and open-ended approach to be used during the interview process (Charmaz, 2017). A reflexive approach was used to code and develop themes from the interviews. This process allows for substantial analytical and interpretive approach by the researchers (Braun & Clarke, 2020). As a process, coding can be acknowledged as a subjective process that obliges the researcher to be reflexive and reflect on their own assumptions and how these can influence their coding (Braun & Clarke, 2020). Thematic analysis offered a flexible approach that allowed the researchers to obtain rich, detailed and complex data. It also allowed for an examination of participants’ varying perspectives, identifying similarities and variation, as well as producing unanticipated thoughts (Nowell et al., 2017).

#### Ethical Issues Pertaining to Human Subjects

Approval for this study was given by Gold Coast University Hospital (HREC/2022/QGC/86553) and Griffith University (2022/573). All participants were informed that their participation in this study was confidential and voluntary, and that they could withdraw their participation at any time. Signed consent forms and interview transcripts were stored, according to Ethical Research Guidelines, on a secure server. Any participant experiencing distress as a result of their participation had the opportunity to access support through the Employee Assistance Program, Beyond Blue, Lifeline, or their General Practitioner.

#### Setting and Sampling Strategy

Four acute mental health units located in public tertiary hospitals in south-east Queensland, Australia were identified as providing care to women between aged 22 to 65 years. Convenience sampling was used to recruit nurse participants from across the four units. Approximately 165 nurses employed on the four units, including nurse managers, clinical nurses, registered nurses, enrolled nurses, and new graduate nurses. Inclusion criteria for participating in this study included being a Registered Nurse or Enrolled Nurse, equivalency of three months or more working in the acute mental health setting, any experience working with women during the pre- and/or postnatal period, and currently employed within one of the acute mental health settings at either hospital. There were no exclusion criteria. Study information was distributed to potential participants via posters, unit newsletters, and direct email to staff via the nurse unit managers. The lead researcher provided brief presentations about the project at staff meetings and nursing shift handovers.

Participants had the option of completing the interview either in person, online (via Microsoft Teams), or by phone. Ten participants completed their interview in person, one participant online, and the final participant by phone. Eleven of the twelve interviews were audio-recorded and transcribed. One interviewee declined to have their interview recorded but was agreeable for the interviewer to take notes during the interview. Interviews ranged in length from 19 min to 58 min, with the average length of interviews being approximately 39 min. Interviews, transcription, and uploading to NVivo for coding and thematic analysis was completed by SL. Coding of the transcripts was identified through review of the transcripts by utilizing an inductive approach. Coding was initially completed by SL, followed by discussion among the three researchers to interpret and refine codes and develop themes and sub-themes.

### Data Analysis

#### Participant Selection

A total of 17 nurses expressed interest (verbally or via email) in participating in this research and were contacted by AM for a screening interview. One did not meet inclusion criteria (unregistered Assistant in Nursing), four were unable to be contacted (after three voicemail messages and three emails), and the 12 remaining nurses consented to participate. A total of twelve nurses were interviewed by SL, and data saturation was achieved after the tenth participant interview.

#### Data Collection

Data collection comprised of a survey for nurses to completed was developed using a university-based research database builder (REDCap) to capture personal demographics including age, gender, parental status, educational and professional background and location (international or domestic), and highest level of nursing education. Additionally, data post-graduate mental health and perinatal education and qualifications, length of nursing and mental health nursing experience and employment status were collected. Survey access was provided through a weblink and QR code that were shared with participants by email and in person via a poster. Demographic results were analysed utilizing descriptive statistics.

The qualitative component of the study involved semi-structured interviews to explore nurses’ experience in providing care to perinatal Consumers^1^, educational experiences and professional development needs related to proving care to pregnant and postnatal women as well as any moral distressing experiences while providing care within an acute mental health setting. The interview schedule was pilot tested with three (non-participant) mental health nurses to identify any issues with the questions which were then refined for clarity for the research process. Once interviews were completed, they were transcribed by SL in preparation for thematic analysis. The authors aligned the thematic analysis with Braun and Clarke's six stages of thematic analysis. These phases start with phase one, the familiarization with the data to immerse deeply into the dataset and identifying initial ideas, patterns, and meanings; phase two, generating preliminary codes to systematically identify and characterise meaningful features across the data; followed by phase three, searching for themes to identify broader patterns by grouping related codes into meaningful themes; phase four, reviewing themes to ensure that each theme is coherent, distinct, and consistent, across the data; phase five, defining and naming themes to refine and finalize each of the themes with assigning meaningful and clear names; and finally, phase 6, and writing the report consisting of a coherent, transparent, and compelling narrative. This process allows for a systemic approach in developing meaningful insights while allowing the researchers to remain engaged in the data (Ahmed et al., 2025).

## Results

### Participant Characteristics

Participant characteristics are summarized in Table 1. Just over half of the participants had completed a university undergraduate degree, or higher, and half had five years or less of clinical experience within a mental health setting. Most nurses were employed as a Registered Nurse or higher.

**Table 1.** Participant characteristics (N = 12).

	**n**	**%**
**Demographics**		
Age (years)		
20–30	5	42
31–40	2	17
41–50	1	8
51–60	3	25
Gender		
Male	2	17
Female	10	38
Children in the Home*		
None	4	36
1–2	6	55
3–4	1	9
**Education**		
Country of Training		
Australia	10	83
Overseas	2	17
Highest nursing qualification*		
Diploma	2	18
Degree	4	36
Masters’	3	27
Doctorate	0	0
Other^1^	2	18
Post-graduate nurse education		
Already completed	7	58
Currently completing	4	33
No	1	8
**Clinical Experience**		
Experience as a nurse (years)*		
0–2	5	45
3–5	1	9
6–10	2	18
11+	3	27
Experience in Mental Health (years)*		
0–2	5	45
3–5	1	9
6–10	3	27
11+	2	18

1 Hospital trained nurses.

*Not completed by one participant.

### Qualitative Data

A total of twelve nurses were interviewed, and data saturation was achieved after ten interviews. Analysis of the nurses’ interview data allowed for the development of four overarching themes: (a) the **nursing care** provided to perinatal Consumers within the acute mental health setting, (b) factors within the **work environment** impacting the nurses’ ability to provide care, (c) **clinical dynamics** between clinical disciplines and Consumers that impact Consumers, and (d) **moral distress** experienced by mental health nurses. Themes and sub-themes can be found in Table 2.

**Table 2.** Themes & Sub-Themes.

Theme	Sub-Themes
4.1 Nursing Care	4.1.1 Advocacy 4.2.1 Empathy & Rapport 4.1.3 Mum & Bub Bonding 4.1.4 Perinatal Education
4.2 Work Environment	4.2.1 Patient Safety in the Context of Aggression 4.2.2 Lack of Resources 4.2.3 Restrictive Environments 4.2.4 Health Care Systems Issues
4.3 Workflow Challenges	4.3.1 Diplomacy 4.3.2 Dynamics Between the Multi-Disciplinary Team 4.3.3 Individual Care vs Group Approach
4.4 Moral Distress	4.4.1 Feelings Experienced by Nursing During Provision of Care 4.4.2 Ethical Situations

### Nursing Care

Nurses reported factors that impacted their ability to provide care to the Consumers admitted to acute inpatient settings. Although they advocated for their Consumers, there were times they were unable to without support from other clinical groups, such as the medical treating team.

#### Advocacy

Nurses identified advocating for Consumers as an important component of their clinical role. However, they felt their advocacy was not always heard by health professionals from other disciplines. For example, participants reported advocating for Consumers with the medical treating team, without success:

And, he wasn’t letting her try, he [Treating Consultant] wasn’t giving her a chance, he wasn’t listening, he was punishing, he was punishing her, always. We’re not… we’re not jail. Like, we’re there to care for them, we’re not there to punish them for anything. So, we tried our best to just advocate for her, but we’re getting much of a… much of the same response that she was getting. So, it was really difficult. (Nurse J)

When nurses experienced difficulties in the advocacy process, they often engaged senior nurses, including the Unit Manager and/or Team Leader, to assist in advocating for Consumers:

We were trying so hard to help this woman and we just couldn’t get anything done for her. And it took me to go up to my Nurse Unit Manager and say “this woman isn’t coping without her baby. You need to go to these doctors because they aren’t listening to me”. And it wasn’t until my Nurse Unit Manager went to them before she had supervised leave with the husband. So… (Nurse D)

While some nurses had some success requesting assistance, some reported they did not always receive the support they were looking for:

I’d escalate this. I’ve been in trouble for doing it before, I’m not afraid to do it. I would definitely be talking to my Team Leader about this and being the Team Leaders I’ve had dealt with up until recently, I’ve found them all to be quite accommodating, and very professional about it. They can obviously see that there's a good rationale behind it and there's good reasoning that this needs to be pursued, regardless, which is good. (Nurse N)

#### Empathy & Rapport

Participants identified the need to have empathy and build rapport with Consumers. Participants identified that listening and providing space for Consumers is important:

…at the basis of it, it's all about using your basics of mental health nursing. So, your communication, your empathy, your therapeutic relationship, using yourself as a tool. And I think when you talk to someone human to human, even if it's something like pregnancy, I think you can get somewhere. You don’t need to know everything, but I think, I’m well prepared because I talked to someone like they’re human, and you put them in the middle of it. (Nurse A)

Participants acknowledged that the women they were caring for may be experiencing complex issues within the community, including domestic violence, substance misuse, and judicial issues resulting in complex care. Despite this, they perceived a connection can be made with the Consumers.

But it is, those, those sort, perinatal health and with them on acute wards, especially you will get the certain patients that really do have a connection with for whatever reason. The care is complicated. They have a whole bunch of other things like domestic violence, and there's drugs, and court cases, and… (Nurse I).

Some also identified complexities impacting the Consumers’ ability to be a parent. The therapeutic relationships between nurses and Consumers are fostered as the nurse acknowledges these complexities.

…as far as I am concerned, every parent, every parent, let me say that again, every parent has an opportunity…to do that too. You’ve got to give that to them. In any way shape or form. Yeah, okay, as parents there's no rule book. We all trip. We all stumble. We all pick ourselves up, and go “That was stupid, I won’t do that again”. And, you move on and learn. So, why is it any different for them? They might just need an extra hand because they’re not feeling good at that particular time, that's fair. (Nurse N)

#### Mum & Bub Bonding

Participants identified the need to facilitate time between the Consumer and her children at home, including infants, as they need time to bond, particularly in the early weeks and months of life.

But even if we’ve had people within the twelve-month cut off…within that twelve month, the engagement has been regarding bonding with the kids, and how we can facilitate that bonding and engagement with the kid, breastfeeding, and how we can help them with that. (Nurse E)

Mental health inpatient admission was acknowledged as potentially impacting mother-infant bonding. For example, the titration of medications within the inpatient setting can negatively impact this bonding time. One participant highlighted this:

That she wasn’t ready. I think that they were adjusting her medications. They like increased her medications slightly because she had schizophrenia and she had relapsed. There was a trigger, which they believed was the birth of the bub, and it was just delayed. And they upped the medication, and they just wanted to make sure there were no adverse side effects. But that woman did not see her baby for two weeks. (Nurse D)

However, nurses reported there were opportunities to provide space for Consumers to spend time with their infants during acute inpatient admissions. Nursing staff were able to facilitate bonding time between a mother and her infant in a safe space within the unit as reported by one participant:

…In this particular ward, we actually had a family room. So, what we did was for the Consumers that were post-pregnancy and newborns, like literally six months or younger that room there the nurse that would be allocated to that Consumer would be in there as a chaperone and the parent, the other parent, if there was one, or other partner, would be there with the child, or the infant and then they would have an interaction with each other, with the child, or the infant. And we would monitor that. And, obviously, if it was obviously something that wasn’t warm and it wasn’t appropriate, well then it would be cut off straight away for the safety of the… you know what I mean. And that worked very well. It was really good. It was so therapeutic. I believe that if that wasn’t in place, I think that the success rate would be lower. (Nurse N)

#### Perinatal Education

The participants spoke about some of the challenges around providing care to women during the perinatal period when they had limited knowledge. They highlighted the limited learning opportunities within their clinical education around perinatal care.

Yeah, now, nothing in particular like if we relate to mental health, no, we didn’t look for that. When it comes to normal studying about medications, and things that you need to take care of, when they are pregnant ladies, pregnant female patients, as patients, there are certain things which we have to take care of especially when it comes to medication management and, what do you call I, when you come to mental health. Like, I did my own bit of research, like, just a bit of read about, like, how to approach psychologically how to, how do you call it, female nurses when they’re in this type of settings. And, it does really help. Yeah, from university, from my study point of view, nothing much… (Nurse L)

Nurses also highlighted that since there is a specialized mother and baby mental health unit within the hospital, nurses within the acute mental health setting are not fully equipped to provide care to this community:

Because we have a specialized unit, I don’t think our ward nurses are very well informed on the ins and outs of perinatal mental health, baby blues to post-partum psychosis. I don’t think we would be very well equipped to pick them up should we even see it. (Nurse E)

Participants highlighted the value of having in-services on the perinatal period. One suggested that perinatal care education should be treated like other education topics:

Having it as a regular in-service just to keep people aware of it, if it was to happen, same as you will, should you be caring for other cases like eating disorders, they’re not always on the unit, but you still have regular education around diagnosis, kind of thing. (Nurse B)

### Work Environment

The acute mental health setting can adversely impact the care that women in the perinatal period experience. Aggression from Consumers directed towards other Consumers, lack of resources (such as accessing breast pumps), and healthcare environments (for instance, staffing factors) that can impact the nurses’ ability to engage in activities beyond Consumer care were all reported by nurses as impacting care.

#### Patient Safety in the Context of Aggression

Nurses were cognizant of aggression and the safety of vulnerable Consumers, including women in the prenatal period. They recognised that women may also be experiencing aggression and violence in the community and that inpatient mental health units could be highly volatile at times, resulting in aggression and/or accidental injury.

Yeah. It's so dangerous, stuff happens all the time, and it could even be by accident by another patient. You don’t want someone to get hurt, and they’re pregnant and I don’t know. Abused, traumatized any more than they may already be or… (Nurse J)

Additionally, another Participant reported that the care required beyond mental health care may be neglected:

Like, we have no idea what's happening. So, they’re put in an environment that's super unsafe, with people that don’t know anything besides the mental health side of it, generally, I assume. And it's just… it's it's…. I just feel the fact that their pregnancy is ignored. (Nurse N)

Admissions into an acute mental health unit for perinatal women can pose risks due to the potential volatility of these units. One Participant noted this contradiction:

Like, we had a patient that was assaulted today, it can happen. Like, they’re with a super volatile community as well. It is their community, they are patients too, but we aren’t keeping them safe in a quiet, nurturing environment. Like, it should be colours, and fluffy bunnies. And something, and we haven’t been trained in any way to know what to look for in anything to do with, you know, pregnancy going wrong. (Nurse J)

#### Lack of Resources

Accessing physical and clinical resources required for proper perinatal care could be limited in mental health wards. When speaking about physical resources, nurses noted the limited antenatal and postnatal care resources their mental health nurses have access to.

I think we do the best we can with the resources we have. So, yeah, I honestly do think we do the best we can. I think we can make more of an effort into moving them to wards where they might benefit a little bit more instead of keeping them on acute ward where it's so low steam and there's no resources, particularly with [our hospital] where we just don’t have any gynaecology what-so-ever. (Nurse D)

Nurses reported having to source these resources, such as breast pumps, from another hospital:

It took me four days the other day to get a breast pump from [another hospital]. And this woman was in so much pain because she couldn’t… she had to manually express in the end. It was terrible. (Nurse D)

Participants also reflected on the clinical resources available. Participants highlighted their need to contact the specialty mom and baby mental health unit when requiring support. One Participant highlighted that other senior clinicians may not be able to provide the support they require.

[Specialty postnatal mental health] nurses are the best resource we’ve got. Whilst she's still pregnant, it's not their speciality technically yet, they are the closest to what we’ve got. Still have the CNC who you would hope that is an area that they know but you can’t guarantee it always. (Nurse D)

Another participant noted that these specialized nurses were able to provide onsite support:

We always…what I’ve seen…we do tend to talk with [specialty postnatal mental health nurses] to get input or get their nurses to come in to monitor something specific if it's been asked or not. (Nurse E)

#### Restrictive Environments

Nurses described the need for Consumers to have restrictive care based on the safety of the foetus, the perceived vulnerability of these Consumers and/or their engagement in care. Sometimes, treatment authorities and/or physical restraints are used to keep the Consumer and the foetus safe. These restrictions impacted the care nurses could provide as this approach to care did not align with the desires of the Consumer.

But the staff having to manage the situation the best we could, keeping the safety of the child in mind. We struggled because you were constantly fighting with a woman who didn’t care if she hurt the baby. Didn’t care what she was doing. Staff had to do what they had to do to keep that baby safe. (Nurse B)

Whereas other times, the restrictions placed on Consumers were seen as being based on the perceived need for care. These restrictions could be through the application of one-to-one specials:

…and it's an inappropriate setting to put them on a ward with a one-to-one, it makes them feel like, you know, they’re in jail and there's a warden, so to speak, staring at them all the time. Or, sitting there staring at them all the time. So, they don’t have that freedom to recover. They’re restricted so much. And, if they’re not restricted, they’re scared the hell out of while being on an acute ward with other people that don’t understand. They don’t get the opportunity to be open, relaxed, and recover. And I think that's where the point is. (Nurse N)

Other times, the restrictions could be through legislation, when a Consumer is placed on a Treatment Authority. This type of restriction sometimes elicited a negative engagement between Consumers and nursing staff, resulting in a delay in rapport building:

Because, she was on a Treatment Authority, and she didn’t want to engage with us. She was, again, at that end of the pregnancy, she felt that…her baby was being threatened in a way, so she was very resistive to nursing staff, and it took us a long time to build a good rapport with her. She fully didn’t trust us and that's really sad to see. Because, you know, we like to put women in boxes. (Nurse A)

#### Health Care System Issues

The healthcare system was viewed as impacting the care Consumers experienced, with influential factors including staffing, time constraints, and a focus on key performance indicators (KPIs). Nurses reported that being understaffed negatively impacted the care that Consumers received. This resulted in some areas of care not receiving optimal attention.

I think it's hard to because like I said before, we are so busy, we’re so… overworked, and understaffed, and under-resourced that you know putting pregnancy as a priority, which we should, and whatever extra monitoring that we have to do, extra research, extra mindfulness, whatever, trying to find the time to do that, day-to-day is really hard. (Nurse J)

Time constraints also impact the care Treating Teams provided to Consumers. The expectations of their patient-loads were viewed as limiting the amount of time spent with Consumers resulting in the potential for adverse reactions from Consumers.

Lack of time. Lack of… support I guess. So, you’ve got like four Treating Teams on our unit, looking after 26, or 24 people. They don’t have time to see their patients on a regular basis, daily, whatever it may be. So obviously patients get annoyed, get distressed, act out, get themselves into these situations. (Nurse C)

One nurse reported that they felt that the organization's need to meet KPIs, such as budgets and Consumer outcomes, carried greater weight than meeting the needs of the Consumers and engaging in a least restrictive practice.

In regards to moving people from PICU [Psychiatric Intensive Care Unit], even the doctors are powerless at that point. The doctors want them to move over, and I feel like, it's said that the organizations more worried about ED [Emergency Department] KPIs. It's a lot about outcomes, budget, and not thinking about actual people, we’re just thinking about numbers, and what it looks like, and crap. (Nurse J)

### Workflow Challenges

Dynamics surrounding clinical care were also reported as affecting the care that nurses provide to Consumers. These dynamics included the diplomacy, and dynamics, between nurses and physicians, and the individualised care provided to Consumers.

#### Diplomacy

Nurses played an integral role in facilitating communication between the medical teams and Consumers. Nurses reported that they navigated challenging conversations and helped Consumers better understand these conversations with, and paperwork received from, the medical teams:

…one more challenge, was making her understand her reports. Like, it's the job of the doctors but you know, sometimes she didn’t get the answers she was expected from the Treating Team. Because, the doctors come in, and they have a chat, twenty minutes, thirty minutes, then they go away. But, if she wanted something in-depth reports and stuff like that, I used to always help her, “this is what it means” and “if you want any clinical advice of things, your doctors are the best people to advise you”. (Nurse L)

Nurses also reported they were often the health professional who provided Consumers with negative news. They spoke about Treating Teams not providing negative news to Consumers due to their concerns of the Consumers’ response. Thus, leaving this task to the nurses to complete.

…the doctors wouldn’t even come out to tell her themselves. They were like “she’ll get aggressive.” No, s**t, so would I. But, so again, they leave it up to us to deliver the, you know, that news. She eventually did, and she did explode. Fair enough. So, we dealt with that. (Nurse J)

One nurse reported that there were times that nurses’ clinical observations and advocacy for Consumers seemed to be unheard by the treating teams.

I had a patient who…like she's been our ward for nine months now, nine months, a very long time. She's in and out unfortunately. She's high risk for AWOL. They finally gave her leave. And she used it so, so well. She came back and was really hoping to get overnight leave because that was the discussion from the doctors – “if you do well, we’ll consider this.” Then in the review, they were like “no, you’re not ready.” And, I was like, “but, she did so well.” And, the right thing to do would be to try to get this lady back home. But, yeah, so, little things like that. So where I get upset with the doctors, but like I don’t make any calls on your plan. Like, I can’t do anything. (Nurse D)

#### Dynamics Between the Multi-Disciplinary Team

The inter-relationship between clinicians and Consumers was also viewed as impacting care outcomes. Nurses reported they spent substantial time with Consumers, but did not feel valued, or heard, by other professional groups.

I think as nurses we spend so much time with them, we spend a lot more time with them than the doctors do. And as nurses we develop a really good relationship with our patients. So, it's hard when you… when you kind of go into reviews really hoping for something for this Consumer and the doctor turns around and goes, “No. No, we’re not doing that. No, you’re not ready.” I’m like, “You’ve seen them once in two weeks, mate.” Like… it's hard. It can be really hard. (Nurse D).

Another participant acknowledged that there are times that they felt dismissed by members of the medical teams due to their role as a nurse.

I believe that… it's possible that from looking at it from a different point of view, that he was dismissing me because I was just a nurse, and he's a Registrar. And, you do get that from time to time. It is what it is. (Nurse N)

#### Individualized Care vs Group Approach

Participants highlighted that although some clinicians aimed to provide individualized care to perinatal Consumers, others acknowledged this did not always happen. One Participant highlighted how easy it could be to be complacent and provide care based on similar, previous presentations, and not based on the uniqueness of each Consumer.

Yeah, I do find that with quite a few nurses that I’ve worked with that. I find that, I found that they just treat every patient as the same approach, you know, like “oh, yeah.” For them, it's a regular thing, or it seems like a regular thing, all drug induced psychosis, or paranoid schizophrenia, bipolar, so this is expected, this is expected, this is expected, this behaviour. (Nurse L)

However, another participant reported that care is not provided based on categorizing Consumers but by what Consumers are working through.

Lack of knowledge, lack of understanding, lack of compassion. I think we all see acute adult mental health as a whole. We don’t separate it into categories of who's dealing with what. (Nurse C)

Another participant reported that instead of getting to the root of the problem for the Consumer, that the psychiatric registrar categorised her into a group, rather than approaching the Consumer as an individual.

He doesn’t want to get down to the root of the problem and he's putting her into a group instead of being treated as an individual. She's being treated in a group of what he's seen before, like, not everyone's an individual. (Nurse I)

### Moral Distress

Experiences of moral distress while providing care to women during the perinatal period was reported by participants.

#### Feelings Experienced by Nurses During Provision of Care

One nurse acknowledged that perinatal health training is not commonly covered in nurse education curricula, making it challenging to safely manage in an acute mental health unit. As a result, when ethical situations arose, this impacted staff, the Consumer, and anyone else observing the situation.

It's not an area that we’re given any sort of training. And even the doctors, at times, are unsure in how we can safely manage the situation. We will be told that we can do one thing but then there will be ethical considerations such as a restraint of a heavily pregnant woman whilst you are able to legally able to do it, in certain situations, the ethical feeling of having people hold down a lady in that position and the impacts that has on both the staff, the lady herself, and also any other witnesses to the event can be very difficult, and how to manage all of that where we stand. (Nurse B)

Another nurse acknowledged worrying about perinatal Consumers on the unit:

I don’t like the fact that it's such a… like I shouldn’t to be the one mainly worrying about my patient being pregnant, like the doctor, like it's “don’t forget she's pregnant”, “what about the…” you know. Like, I feel like the extra stress on us as nurses, cause we’re the ones, we’re not midwives, we’re not, you know, specialized in babies or pregnancy in any way. (Nurse J)

Another nurse reported feeling like a “midwife” at times as they provided care to women in the perinatal period.

Several patients, you have to set a limit, you draw a line, of course, that was the same with this patient, as well. But, being pregnant, we just have to, we as nurses have to be a bit more generous with some things. Especially when we are caring for her. To show her that “we care for you and your unborn… and we want you to really improve and look forward to your delivery.” Which is good. So, at times, at times I felt like a midwife as well [laughter]. (Nurse L)

Finally, another nurse reported struggling, at times, with caring for these Consumers, and found it difficult to find an outlet:

I struggle sometimes. It's… I’ve really had to find an outlet. I think I’ve learned not to really hold on to them and take them to heart. I used to and then I just got too upset. (Nurse D)

#### Ethical Situations

Nurses acknowledged that ethical situations arose when a pregnant woman was admitted to an acute mental health unit. One participant highlighted the conflict between the involvement of child protection services as a result of the Consumers’ social circumstances, such as intravenous drug use, domestic violence, or mental illness. However, despite all of this, they recognised that the Consumer had immense love for their baby and still wished to raise the baby, once born, despite her current life challenges.

Ummh, yeah. So a big one is child protection. Obviously we work with a lot of marginalized communities. We work with IV drug users, we work with women who may be in DV relationships, women who have schizophrenia, bipolar which may affect their ability to parent their child and I think as much as you want your baby to be with their child, or have a healthy pregnancy, you know it's not always going to be that way and it's so sad. Especially when you see that the mom really wants their baby or loves their baby. (Nurse A)

Another nurse reported challenges in advocating for their Consumer. They felt that the direction they were being given by the treating team conflicted with what they felt was the right thing to do.

But, one thing I did for my patient who I was discussing in earlier responses was [that] there was one time where there was, there was a bit of medication issue and ethically or morally that the doctors asked me to give that medication, because from the doctors experience, they felt that it was safe to give in that, what do you call time difference, you know every medication has a four hour gap, six hour gap, or whatever. (Nurse L)

This nurse further reported feeling alone while navigating medication safety for a pregnant patient.

No, I felt that it was challenging, definitely. Because I was generally calling in with the doctors, I felt alone with that thing. (Nurse L).

## Discussion

This research aimed to explore mental health nurses’ perceptions and experiences, including their experiences of moral distress, related to providing care to women during the perinatal period within acute mental health settings. Themes arising from analysis of nurses’ interviews highlighted those areas that impacted moral distress included nursing care, work environment, and clinical dynamics. Nurses reported not feeling heard by the other clinical disciplines and not always experiencing support when advocating for their Consumers. This is congruent with other research in which mental health nurses have also reported that ethical concerns they have raised are ignored by authorities (Tavakol et al., 2023) or not given equal consideration despite nurses spending more time with the Consumers than other clinical disciplines (Deady & Mccarthy, 2010), creating anxiety for nurses around sharing their concerns, and thus triggering moral distress (Tavakol et al., 2023). Additionally, some nurses reported communication challenges, particularly around delivering bad news to Consumers. Nurses are considered valued members of the clinical team and can play an important role in conveying bad news. However their role in actually delivering the bad news has not been well researched (Abbaszadeh et al., 2014).

Nurses conveyed their concerns for, and compassion and empathy in how they cared for, vulnerable Consumers, including women during the perinatal period. Nurses reported that empathy improved communication between nurses and Consumers, resulting in enhanced quality of care (Usenmez & Gumus, 2021). Providing compassionate care involved diverse actions and values. Engaging in compassionate care respects the dignity of Consumers and is intertwined with nursing competencies (Tehranineshat et al., 2019).

Consumer safety is twofold for mental health nurses, acknowledging aggression risks within the Consumers’ personal life and within the clinical setting. Primary care providers, including nurses, play a significant role in protecting and promoting women's health as well as detecting and preventing serious consequences of domestic violence (Simsek-Cetinkaya & Evrenol Ocal, 2023). Nurses in the present study acknowledged that women in this community were more likely to experience domestic violence, substance misuse, and judicial issues which resulted in increasingly complex care, however, they were still able to build rapport with these women and families.

Nurses also acknowledged the potential aggression risks within the acute mental health settings. In alignment with previous research, the nurses within this study viewed safety as being of the utmost importance and used safety to underpin their clinical interventions and judgement (Slemon et al., 2017). Furthermore, these nurses were able to employ empathy as they built these therapeutic relationships, which is also indicative of research that has found that health professionals are mindful in how they respond to women with these lived experiences in a gender-sensitive, and safe, approach (O'dwyer et al., 2019). Mothers bonding with their babies is crucial to the development of a strong mother-infant relationship, and attachment is a key developmental process for infants (Phuma-Ngaiyaye & Kalembo, 2016). These processes allow mothers and infants to establishing a nurturing connection in which infants can signal their needs to their mother and have their needs met, which is vital for the infant's health and development (Phuma-Ngaiyaye & Kalembo, 2016). However, the mother-infant contact that facilitates normal attachment and bonding processes may not be accommodated on acute mental health units due to the potential for physical aggression on the unit. Participants in this study highlighted that aggression within the acute mental health setting, even if accidental, may be unavoidable and may require a one-to-one special to ensure safety. One-to-one specials are often used within mental health settings to monitor, and manage, consumers’ behaviours through close supervision of one consumer by one mental health nurse to minimize risk to the Consumer or to others (Reen et al., 2020). Research has found that between 25 and 80 percent of nurses reported experiencing patient violence, and it is expected that these rates are under-reported (Stevenson et al., 2015).

Previous research into moral distress among mental health nurses have highlighted sources of moral distress as enforced medication adherence, physical restraints, use of seclusion rooms, mandatory admissions, enforced electroconvulsive therapy (ECT), and involvement in group therapy (Tavakol et al., 2023). The participants in this study also reporting having to utilize restraints with pregnant women. Participants reported struggling with keeping the baby safe during pregnancy when the Consumer engaged in activities that were potentially harmful to development of the foetus. Participants highlighted the need to utilize restrictive practices to keep these Consumers and their foetuses safe, including having the Consumer placed on a treatment authority, restraining the Consumer to administer medication, or on a one-to-one specialling when constant supervision and care was required.

It is acknowledged that experiencing a degree of moral distress may have positive effects, including highlighting moral obligations and dilemmas and helping to maintain high standards of care (Jansen et al., 2022a). However, moral distress can negatively affect the delivery of healthcare and the wellbeing of healthcare clinicians themselves (Jansen et al., 2022a) and can result in negative feelings including sadness, powerlessness, bad conscience, guilt, shame, emotional numbness, despondency, cynicism, and resignation (Jansen et al., 2022b). There are two potential outcomes when nurses experience moral distress. First, nurses may leave the nursing profession altogether. Second, nurses remain in nursing, but become emotionless to patients’ suffering and do not engage emotionally. In both scenarios, patient care becomes compromised (Tavakol et al., 2023). Although the participants did not explicitly report that they planned to leave the nursing profession, they did highlight their frustrations and experiences of moral distress. Nurses reported experiencing sadness related to a lack of trust from Consumers and the necessity of involving Child Protective Services when babies were deemed to be at risk.

Finally, our results suggest that there is currently a gap the training offered to mental health nurses in providing care to perinatal patients, particularly within mental health settings. Nurses reported that perinatal care was not covered within their undergraduate nursing programs and not covered within the context of mental illness within post-graduate mental health studies. The opportunity for nurses to engage in professional development to enhance their competencies and foster quality and safe care is paramount (Legere et al., 2017). Nurses need to be able to demonstrate evidence-based knowledge, attitudes, judgement, and skills when considering illnesses that may have serious consequences for women and their babies (Legere et al., 2017).

## Conclusion

These findings highlight the potential risks of having women admitted to acute mental health units during the perinatal period, including interrupted bonding between mother and baby, limited perinatal education and resources for mental health nurses, and challenging clinical dynamics. In a context where ethical situations regularly arise, negative feelings associated with moral distress, including worrying and struggling, were highlighted by nurses and impacted care provision. However, despite these challenges, the research highlighted that mental health nurses engaged in compassionate care towards pregnant women and families and demonstrated advocacy, reflected upon challenging conversations, and were cognizant of patient safety. It is important for nurses to feel supported to navigate these challenges and to develop the skillsets required to provide holistic care for their Consumers.

## Notes

1. Consumer will be used throughout, rather than Patient
